# Correlational research on college students’ physical exercise behavior, academic engagement, and self-efficacy

**DOI:** 10.3389/fpsyg.2025.1428365

**Published:** 2025-02-03

**Authors:** Wei Gao, Jiaxin Chen, Zhi Tu, Ming Li

**Affiliations:** ^1^School of Physical Education and Sport Science, Fujian Normal University, Fuzhou, China; ^2^Fujian Forestry Vocational Technical College, Nanping, China

**Keywords:** college students, physical exercise, level of learning engagement, autonomous efficiency, positive self-confidence

## Abstract

**Objective:**

This study aims to investigate the current status of physical exercise behavior, academic engagement, and self-efficacy among non-physical education college students. Additionally, it sought to analyze the relationships between these factors in order to explore the potential impact of physical exercise on learning and self-efficacy.

**Methods:**

To examine the current status and relationships between physical exercise behavior, academic engagement, and self-efficacy, this study used the Physical Activity Rating Scale (PARS-3), the Utrecht Work Engagement Scale (UWES), and the General Self-Efficacy Scale (GESE) as research tools. A stratified random sampling method was employed, and non-physical education college students were selected as participants. A total of 1,596 valid questionnaires were analyzed. The data were processed using SPSS 26.0, AMOS 26.0, and Excel 2010, with statistical analyses including one-way ANOVA, correlation analysis, regression analysis, and mediation effect testing.

**Results:**

Physical exercise behavior among non-physical education students showed significant positive correlations with academic engagement (*r* = 0.207, *p* < 0.01) and self-efficacy (*r* = 0.218, *p* < 0.01). Academic engagement was also strongly positively correlated with self-efficacy (*r* = 0.811, *p* < 0.01). The partial mediating effect of physical exercise on academic engagement was significant, with the mediation ratio of ab/c = 84.7%.

**Conclusion:**

Physical exercise behavior significantly predicts academic engagement, and a positive predictive relationship exists between physical exercise and self-efficacy. Additionally, self-efficacy plays a significant role in predicting academic engagement. Self-efficacy partially mediates the relationship between physical exercise behavior and academic engagement.

## 1 Introduction

Moderate physical exercise can enhance physical fitness and promote overall health, playing a significant role in college students’ physical and mental development. Research has shown that regular physical activity improves bodily functions and mental health and has a positive effect on alleviating anxiety among university students (Reiner et al., 2013; Zubala et al., 2017; Lin and Gao, 2023). In addition, physical exercise not only benefits physical health but also promotes cognitive function, thereby influencing an individual’s learning and behavioral performance. Neuroscientific research has provided evidence of a strong connection between exercise and cognition, highlighting the positive effects of physical activity on cognitive development (Castelli et al., 2014; Erickson et al., 2015; Mandolesi et al., 2018). Physical activity triggers the release of neurotransmitters that are beneficial for learning and memory, and integrating exercise into classroom and/or learning environments can promote cognitive function (Doherty and Forés Miravalles, 2019). These neurotransmitters include dopamine, which is associated with motivation, attention, and learning, and serotonin, which enhances mood, and norepinephrine, which improves attention, perception, and drive (Basso and Suzuki, 2017). Therefore, regular physical exercise and high cognitive function are crucial for overall health (Gopinath et al., 2018). Exercise also enhances students’ self-discipline, thereby increasing their academic engagement and promoting improved academic performance. Research has shown that physical exercise has a positive impact on academic outcomes, while reduced physical activity can lead to a decline in academic performance (Howie and Pate, 2012).

Additionally, studies indicate that exercise can activate molecular pathways, effectively improving the brain’s molecular mechanisms involved in complex skill learning. Physical activity regulates the cognitive learning functions of the nervous system, enhancing memory and learning performance (Chen et al., 2019). Thus, physical exercise can influence the level of academic engagement to some extent.

Academic engagement refers to a positive, fulfilling psychological state related to learning, which encompasses three dimensions: vigor, dedication, and absorption. It reflects the effort that students invest in the learning process and is closely related to improvements in their academic performance (Schaufeli et al., 2002a,b). From the perspective of measurements and influencing factors, academic engagement is seen as the energy and effort students invest in their learning, which can be observed through behavioral, cognitive, and emotional indicators (Bond, 2020). Recent studies have also highlighted the significant role of academic engagement in academic success, showing a strong correlation between engagement and factors such as learning persistence, academic satisfaction, academic performance, and completion rates (Xie et al., 2020; Kuh, 2009). From the perspective of research on academic engagement, it is emphasized that it is not only a multifaceted concept but also a context-dependent one. The value orientation of academic engagement may vary across different contexts, such as social institutions, schools, classrooms, and learning activities (Skinner and Pitzer, 2012). Additionally, it has been found that during the learning process, an individual’s goal-directed behavior is influenced by self-efficacy, which constrains their level of effort in different ways. Higher self-efficacy expectations lead individuals to exert greater effort to achieve their goals (Wu et al., 2020).

Research has shown that active physical exercise participation significantly enhances college students’ self-efficacy (Zhang, 2015; Baghbani et al., 2023). It has also been emphasized that there is a strong and stable relationship between physical activity and self-efficacy (Bandura, 2004). Physical exercise has a considerable impact on improving an individual’s learning ability, self-confidence, and social skills, thereby boosting motivation and efficiency in learning. It promotes the organic development of physical exercise, cultural learning, and social integration. In sports research, participation in physical activities is considered one of the primary sources of efficacy information, as it influences self-efficacy by providing experiences of mastery or achievement (McAuley et al., 2011). Therefore, practitioners can enhance self-efficacy through well-designed exercise programs, highlighting the strong correlation between self-efficacy and perseverance in physical exercise and various sports. Those with higher self-efficacy tend to demonstrate greater consistency in their physical exercise routines.

As previously described, Bandura’s social learning theory views human functioning as a “triadic reciprocal model, where behavior, cognition, personal factors, and environmental factors are all interacting determinants” (Bandura, 1986). This new theory has provided significant advancements in both the fields of psychology and organizational behavior. In exploring the relationship between academic self-efficacy and academic engagement, it was found that when students have low academic self-efficacy, their motivation to learn is insufficient, and they are unwilling to invest time in studying. In contrast, students with high academic self-efficacy are highly motivated and willing to devote more energy to their learning (Bassi et al., 2007). Other studies have also shown that the higher the self-efficacy, the greater the effort and the better the performance in sports (Qin et al., 2013). Based on this, we propose the following theoretical hypotheses:

*H1:* Physical exercise behavior positively predicts academic engagement.

*H2:* There is a positive correlation between physical exercise behavior and self-efficacy.

*H3:* Self-efficacy significantly positively predicts academic engagement.

## 2 Research methods

### 2.1 Participants

This research focused on college students from non-physical education majors across many universities. Using a stratified random sampling method, questionnaires were distributed to 1,800 students to investigate their current levels of academic engagement, physical exercise, and self-efficacy.

### 2.2 Research procedure

This study strictly adhered to ethical guidelines to ensure the protection of participants’ rights and privacy. First, prior to conducting the survey, we provided detailed information about the study’s purpose and procedures to all participating teachers and obtained approval from the school. Subsequently, all participating students signed informed consent forms, ensuring that they were fully aware of the study’s objectives, the voluntary nature of their participation, and their right to withdraw at any stage. Additionally, the research team emphasized that all collected data would be kept confidential, with all questionnaires processed anonymously to minimize the risk of social desirability bias. Participants’ personal information was kept confidential and used exclusively for research purposes. Throughout the survey process, the researchers ensured that participants were aware they could withdraw at any time without any negative consequences.

### 2.3 Physical activity rating scale (PARS-3)

The Physical Activity Rating Scale (PARS-3) (Liang, 1994) has three items, mainly measuring the amount of physical exercise from the intensity, time, and frequency of exercise. The 5-point Likert scoring method is mainly used to measure the physical activities of college students. The higher the score, the greater the amount of physical exercise. The reliability test of the questionnaire can reflect the consistency of the internal structure of the questionnaire well.

After processing the recovered questionnaires with SPSS 26.0, the Cronbach’s alpha coefficient for the Physical Exercise Rating Scale (PARS-3), as shown in Table 1, is 0.600. This coefficient suggests that while the scale’s reliability is moderate, it remains acceptable. The consistency and stability of the items are satisfactory, which supports their use in this study.

**Table 1:** Confirmatory factor analysis versus model comparison (*N* = 1,596).

Model	X^2^	df	AIC	BIC	RMSEA	CFI	TLI	SRMR
M1	1380.968	404	1502.968	1830.859	0.039	0.979	0.977	0.019
M2	1522.609	378	1696.609	2164.257	0.044	0.975	0.972	0.022
M3	6468.310	405	6588.310	6910.825	0.097	0.869	0.860	0.058

AIC, Akaike Information Criterion; BIC, Bayesian Information Criterion; RMSEA, Root Mean Square Error of Approximation; CFI, Comparative Fit Index; TLI, Tucker-Lewis Index; SRMR, Standardized root mean squared residual.

### 2.4 Utrecht work engagement scale-student (UWES-S)

The Utrecht Work Engagement Scale for Students (UWES-S), developed by Schaufeli et al. (2002a,b), is specifically designed to measure students’ academic engagement. It has 17 items distributed across three dimensions: vigor (questions 1–6), dedication (questions 7–11), and concentration (absorption) (questions 12–17). The scale uses a 7-point Likert scoring system to calculate the total score for assessing academic engagement. The collected data were analyzed using SPSS 26.0. The Cronbach’s alpha coefficient for the UWES-S was 0.974, indicating very high reliability. The alpha coefficients for the vigor, dedication, and absorption dimensions were 0.936, 0.933, and 0.935, respectively, demonstrating excellent consistency and stability of the items, thus supporting the validity of this study.

### 2.5 General self-efficacy scale (GESE)

The General Self-Efficacy Scale (GESE) was developed by Schwarzer, a German scholar (Schwarzer and Jerusalem, 1995). This scale has 10 items, using Likert’s 4-grade scoring method, and only the total score is considered for evaluation. A high score indicates high self-efficacy. After the questionnaire was recovered and processed using SPSS 26.0, the Cronbach’s *α* coefficient for the GSES was found to be 0.944, indicating excellent reliability of the items, which supports the robustness of this study.

### 2.6 Statistical analyses

To analyze the data, we utilized SPSS 26.0, AMOS 26.0, and EXCEL 2010. We assessed the reliability of the questionnaire through exploratory factor analysis and verified its validity using confirmatory factor analysis. Compare the differences between different sexes in each variable by t-test, Differences among variables based on gender were evaluated using t-tests, while differences between academic grades and majors were examined with F-tests. Pearson correlation was employed to explore the relationships between variables. The mediating effect of self-efficacy between physical exercise behavior and academic engagement was analyzed using Hayes’ mediation analysis procedure. This approach allowed for an objective analysis of the influencing paths and mechanisms.

## 3 Results and analysis

### 3.1 Common method deviation test

Common method bias refers to the artificial covariance between predictor and criterion variables caused by using the same data source or raters, similar measurement environments, item context, or characteristics of the items themselves (Zhou and Long, 2004). Since the scales in this study were self-reported by the same participants, there may be a potential for common method bias, which requires testing for its presence. In this study, we used Harman’s single-factor test (Xiong et al., 2012) and the two-factor model method (Podsakoff et al., 2012) to assess the severity of common method bias in the research.

As shown in Table 1, in the two-factor model, we first constructed a one-factor baseline model (M1) for the three study variables using confirmatory factor analysis, followed by a two-factor model (M2) that incorporated the method factor. We then compared the fit indices of the two models (M2-M1) and found that ΔCFI = −0.004, ΔTLI = −0.005, ΔRMSEA = 0.005, and ΔSRMR = 0.0003. There was no significant improvement in the fit indices, and the Standardized Root Mean Squared Residual (SRMR) remained virtually unchanged. The added method factor did not improve the model, confirming no significant common method bias. In Harman’s single-factor analysis (M3), when all indicators were combined into a single common factor for fitting, the fit indices were χ^2^/df = 15.971, CFI = 0.869, TLI = 0.860, RMSEA = 0.097, and SRMR = 0.0581. All indices were far from the critical thresholds and were considerably worse than those of the baseline model (M1). Therefore, there is no evidence of severe common method bias or variance.

### 3.2 Descriptive statistical analysis

The average total score for physical exercise among college students was 26.92, with a standard deviation of 20.13. These findings suggest moderate physical exercise levels among non-physical education students, with strong awareness of and motivation for exercise. The mean values for exercise intensity, duration, and frequency were 3.1, 3.4, and 3.21, respectively, exceeding the critical value of 3. This suggests that non-physical education students engage in physical exercise to a certain extent.

To gain a comprehensive understanding of the academic engagement of non-physical education students, we primarily used descriptive statistics to analyze their overall academic engagement and the basic characteristics of the three dimensions. As shown in Table 2, the overall mean score for academic engagement was 4.43, which is above the theoretical value of 3, indicating that the academic engagement level of non-physical education students is slightly above average. In terms of the dimensions, the vigor dimension had the highest mean score of 4.53, followed by the dedication dimension, with a mean score of 4.39, and the absorption dimension, which had a mean score of 4.35, ranking third. The average score for general self-efficacy was 2.76 ± 0.81, indicating that the general self-efficacy level of non-physical education students is moderate.

**Table 2:** Descriptive statistical analysis of each study variable.

	Min	Max	Mean	SD	Median	Skewness	Kurtosis
Total score of physical exercise	0.00	100.00	26.92	20.13	24.00	0.97	0.24
Exercise intensity	1.00	5.00	3.10	0.92	3.00	0.23	−0.59
Exercise time	1.00	5.00	3.40	0.97	3.00	0.05	−0.94
Exercise frequency	1.00	5.00	3.21	0.96	3.00	0.16	−0.65
Utrecht work engagement	1.00	7.00	4.43	1.53	4.88	−0.58	−0.98
Vitality dimension	1.00	7.00	4.53	1.53	5.00	−0.62	−0.74
Dedication dimension	1.00	7.00	4.39	1.61	4.80	−0.50	−0.98
Focus dimension	1.00	7.00	4.35	1.60	4.83	−0.46	−1.08
Self-efficacy	1.00	4.00	2.76	0.81	2.90	−0.26	−1.33

### 3.3 Correlation analysis

The Pearson correlation coefficients and significance levels for physical exercise behavior and its items, academic engagement and its dimensions, and self-efficacy were tested. The data in Table 3 show the following results:

1. There is a significant positive correlation between physical exercise behavior and academic engagement among non-physical education students (*r* = 0.207, *p* < 0.01), with a weak correlation. This indicates that the stronger the physical exercise behavior, the higher the level of academic engagement. In terms of the individual items of physical exercise behavior, the correlation coefficients between exercise intensity, duration, and frequency with academic engagement are 0.171, 0.163, and 0.116 (*p* < 0.01), respectively, suggesting significant positive correlations. The correlations between physical exercise behavior and its items with vigor are 0.201, 0.157, 0.153, and 0.123 (*p* < 0.01), showing significant positive correlations. Similarly, the correlations between physical exercise behavior and its items with dedication are 0.208, 0.171, 0.169, and 0.119 (*p* < 0.01), indicating significant positive correlations with dedication. Finally, the correlations between physical exercise behavior and its items with absorption are 0.195, 0.170, 0.156, and 0.098 (*p* < 0.01), demonstrating significant positive correlations.
2. There is a significant positive correlation between physical exercise behavior and self-efficacy among non-physical education students (*r* = 0.218, *p* < 0.01), with a weak correlation. Regarding the individual items of physical exercise behavior, the correlation coefficients between exercise intensity, duration, and frequency with self-efficacy are 0.189, 0.170, and 0.095 (*p* < 0.01), all showing significant positive weak correlations.
3. There is a significant positive correlation between academic engagement and self-efficacy (*r* = 0.811, *p* < 0.01), with a strong correlation. The dimensions of academic engagement—vigor, dedication, and absorption—also show significant positive correlations with self-efficacy, with correlation coefficients of 0.788, 0.776, and 0.794 (*p* < 0.01), respectively, reflecting strong correlations. In summary, there are significant correlations between the three variables—physical exercise behavior, self-efficacy, and academic engagement—among non-physical education students. These findings provide a foundation for further testing the mediating effects among the variables.

**Table 3:** Correlation analysis among study variables (*N* = 1,596).

	Physical exercise	Exercise intensity	Exercise time	Exercise frequency	Utrecht work engagement	Vitality	Dedication	Focus	Self-efficacy
Physical exercise	1								
Exercise intensity	0.744**	1							
Exercise time	0.787**	0.451**	1						
Exercise frequency	0.677**	0.354**	0.338**	1					
Utrecht work engagement	0.207**	0.171**	0.163**	0.116**	1				
Vitality	0.201**	0.157**	0.153**	0.123**	0.966**	1			
Dedication	0.208**	0.171**	0.169**	0.119**	0.972**	0.909**	1		
Focus	0.195**	0.170**	0.156**	0.098**	0.973**	0.900**	0.929**	1	
Self-efficacy	0.218**	0.189**	0.170**	0.095**	0.811**	0.788**	0.776**	0.794**	1

*At 0.05 level (two tails), the correlation is significant. **At 0.01 level (two-tailed), correlation is significant.

Therefore, under the assumption that all variables have been centered, the following regression equations can be used to describe the relationships between the variables:

(1) Y=cX+e1

(2) M=aX+e2,

(3) $$Y=c^{\prime }X+bM+e3$$

where the coefficient c in Equation 1 represents the total effect of the independent variable X on the dependent variable Y, the coefficient *a* in Equation 2 represents the effect of the independent variable X on the mediator variable M, and the coefficient *b* in Equation 3 represents the effect of the mediator M on the dependent variable Y after controlling for the effect of X. The coefficient *c*′ in Equation 3 represents the direct effect of the independent variable X on the dependent variable Y after controlling for the mediator M. The residuals e1~e3 represent the errors of the respective regressions (Mackinnon et al., 1995). In this simple mediation model, the mediation effect is equal to the indirect effect, which is the product of the coefficients abab, and it is related to the total effect and the direct effect through the following Equation 4 (Baron and Kenny, 1986):

(4) $$c=c^{\prime }+\mathrm{ab}$$

**Figure 1:**
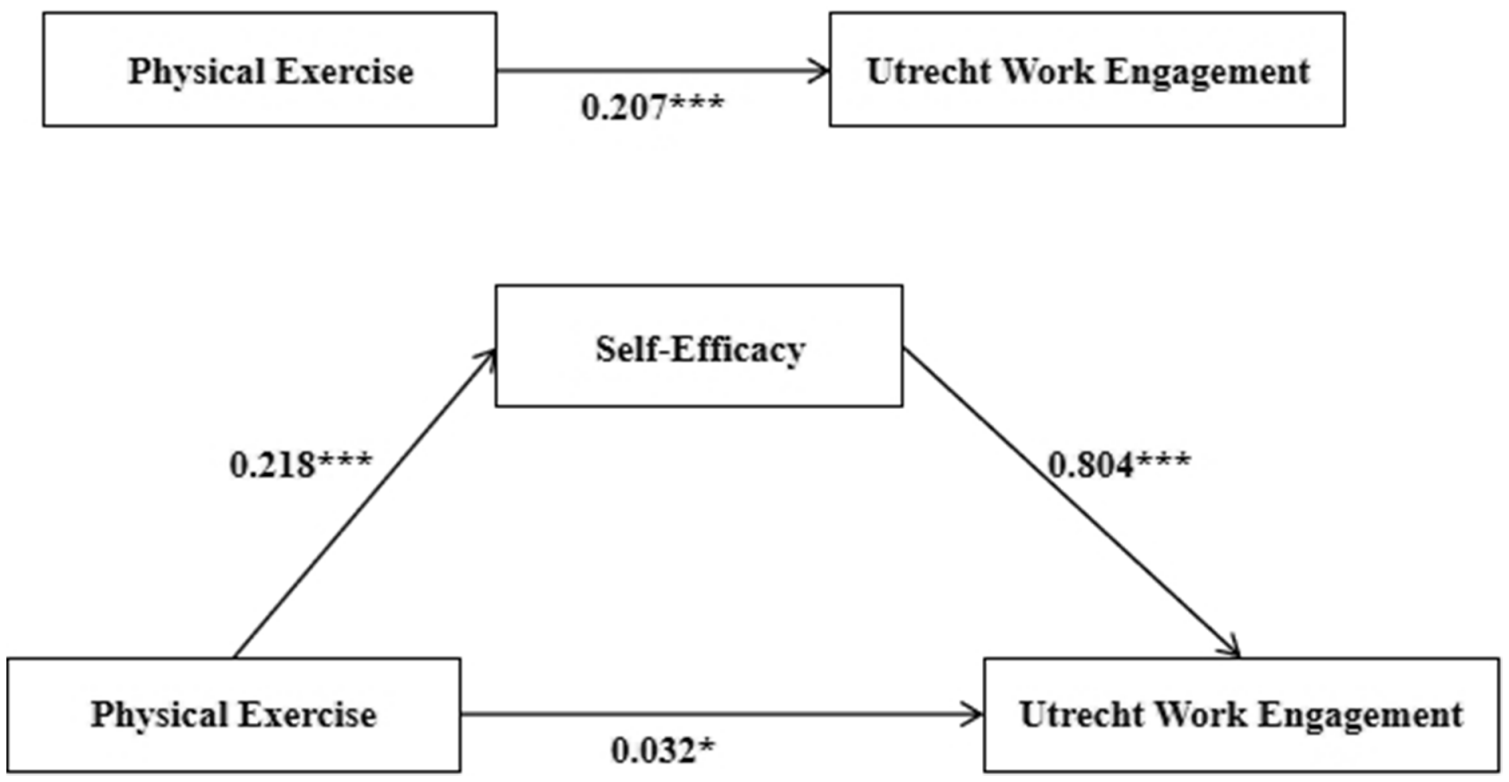
Path diagram for mediating effect test.

As shown in Table 4, the first step was to test coefficient c. In Equation 1, *β* = 0.207 and *p* < 0.001, indicating that c = 0.207, which supports the mediation effect. In Equation 2, the coefficient a was tested, with β = 0.218 and *p* < 0.001, resulting in a = 0.218. In Equation 3, the coefficient for self-efficacy’s effect on academic engagement was β = 0.804 and *p* < 0.001, meaning b = 0.804. Both models showed significance, confirming that the indirect effect is significant. Moving directly to step 4, in Equation 3, the coefficient for the direct effect of physical exercise behavior on academic engagement was β = 0.032 and *p* < 0.05, indicating that the direct effect (c’) is significant. Since the signs of a, b and c are the same, it suggests that the partial mediation effect of physical exercise behavior is significant. Furthermore, ab/c = 0.218 * 0.804 / 0.207 = 84.7%, indicating that the mediation effect accounts for 84.7% of the total effect. The mediation effect path diagram is shown in Figure 1.

**Table 4:** Stepwise regression method for mediating effect test.

Equation	Equation 1		Equation 2		Equation 3	
	Utrecht work engagement		Self-efficacy		Utrecht work engagement	
	β	t	β	t	β	t
Physical exercise	0.207(c)	8.451***	0.218(a)	8.9021***	0.032(c’)	2.144*
Self-efficacy					0.804(b)	53.556***
R square	0.043		0.047		0.658	
Adjusted R square	0.042		0.047		0.658	
F	71.42***		79.254***		1534.062***	

Note: *P*<0.001***; *P*<0.05*.

To further test the mediation effect pathway, Preacher and Hayes (2004) proposed several arguments that facilitate the estimation of the indirect effect of the independent variable (X) on the dependent variable (Y) through the mediator (M). The indirect effect was formally tested for significance using the bootstrap method in the PROCESS plugin for SPSS. This method provides outputs related to the evaluation of mediation. In this analysis, physical exercise was considered the independent variable (X), self-efficacy as the mediator (M), and academic engagement as the dependent variable (Y). Model 4 was selected, with 5,000 bootstrap resamples. The output was checked to see if the 95% confidence interval included zero. The mediation effect was considered significant if the confidence interval did not contain zero. The results are shown in Table 5.

**Table 5:** Bootstrap mediating effect test results.

Effect relationship	Effect value	SE	LLCI	ULCI	Effect proportion
Total effect	0.0158	0.0019	0.0121	0.0194	
Direct effect	0.0025	0.0011	0.0002	0.0047	16%
Indirect effects	0.0133	0.0014	0.0104	0.0162	84%

The results indicate that the total effect has an effect size of 0.0158, with a 95% bootstrap confidence interval of [0.0121, 0.0194], which does not include zero, confirming that the total effect is statistically significant. The direct effect has an effect size of 0.0025, with a 95% bootstrap confidence interval of [0.0002, 0.0047], which also does not include zero, indicating that the direct effect is statistically significant. This means that the direct effect of physical exercise (the independent variable) on academic engagement (the dependent variable) is 0.0025, accounting for 16% of the total effect. The indirect effect has an effect size of 0.0133, with a 95% bootstrap confidence interval of [0.0104, 0.0162], which does not include zero, confirming that the indirect effect is statistically significant. This suggests that the indirect effect of physical exercise on academic engagement through the mediator, self-efficacy, is 0.0133, accounting for 84% of the total effect, further confirming that the mediation effect represents 84% of the total effect. Since the confidence interval for the direct effect of physical exercise on academic engagement still does not include zero after including the mediator, it can be concluded that self-efficacy plays a partial mediating role in the relationship between physical exercise and academic engagement.

## 4 Discussion

Students with high self-efficacy tend to perform better and exhibit greater persistence when facing new and challenging situations (Bandura, 2001). Related studies have shown that the more actively individuals engage in physical exercise, the higher their self-efficacy (Anderson and Feldman, 2020; Han et al., 2022). This is consistent with our findings, which reveal a significant positive correlation between physical exercise behavior and self-efficacy. Building on this finding, physical exercise behavior is not only closely linked to self-efficacy but may also influence students’ academic performance by enhancing their self-efficacy. Previous research has shown a strong correlation between physical exercise and students’ academic performance (Li et al., 2021), which aligns with our results.

Correlation analysis indicates that physical exercise behavior is significantly positively correlated with academic engagement among non-physical education students in Fuzhou, and a positive correlation was also found in the dimensions of focus, vitality, and dedication, with all correlation coefficients being positive. Further regression analysis shows that physical exercise behavior can positively predict the level of academic engagement. Recent studies have also reported a positive correlation between physical exercise and students’ academic performance, which supports our findings (Cid and Muñoz, 2017).

Both physical exercise behavior, exercise intensity, duration, and frequency are significantly positively correlated with general self-efficacy. Further regression analysis indicates that physical exercise behavior has a significant positive predictive effect on self-efficacy. This suggests that physical exercise can enhance an individual’s confidence and coping ability in response to social challenges (Zhu, 2021; Wu et al., 2022). Other studies have also highlighted that self-efficacy plays a crucial role in motivating individuals to engage in academic activities, a process that is essential for achieving academic success (Honicke and Broadbent, 2016).

### 4.1 Medium relational

The results of the bootstrap mediation effect analysis show that the total effect of general self-efficacy on academic engagement is 0.0158 (*p* < 0.05), with a direct effect of 0.0025 (*p* < 0.05) and an indirect effect of 0.0133 (*p* < 0.05). This indicates that general self-efficacy plays a partial mediating role in the relationship between physical exercise behavior and academic engagement among non-physical education students in Fuzhou. Thus, two pathways are identified in this study: one is the direct path where physical exercise behavior directly influences academic engagement, i.e., “Physical Exercise Behavior → Academic Engagement”; the second is the indirect path where physical exercise behavior influences academic engagement through the mediating effect of general self-efficacy, i.e., “Physical Exercise Behavior → General Self-Efficacy → Academic Engagement.”

Social Cognitive Theory (SCT) posits that self-efficacy influences intermediary structures, participation intentions, and goal setting in physical activities, which are crucial for supporting changes in health behaviors (Bandura, 2004). SCT also suggests that humans possess extraordinary symbolic abilities that successfully respond to challenges, adapt, and modify their environments (Stajkovic and Luthans, 1998). After setting goals, individuals invest more time and effort, improving efficiency and contributing to better academic performance. These findings provide valuable insights for future intervention studies, supporting using SCT to alter physical exercise behaviors and enhance self-efficacy (Silveira et al., 2020).

### 4.2 Practical significance

First, college students generally self-determine their physical exercise, which stimulates intrinsic motivation and fosters task completion. As a result, students gain confidence in other aspects, such as their performance and concentration in learning. This process fulfills students’ basic psychological needs, enhancing their self-confidence and perseverance. Consequently, they invest higher levels of enthusiasm and interest in their studies, increasing academic engagement. Second, when students maintain a high level of self-efficacy, they believe they have the ability and confidence to face challenges and difficulties in learning, committing themselves fully to the learning process. Self-efficacy influences the choice of personal behavior; individuals with high self-efficacy are more likely to choose challenging tasks, remain active despite setbacks, and stay prepared to ensure success (Ouweneel et al., 2011). Therefore, general self-efficacy plays a positive role in academic engagement, directly influencing students’ academic involvement. Finally, this study concludes that both physical exercise behavior and general self-efficacy are key factors affecting academic engagement. Furthermore, general self-efficacy plays a partial mediating role—only a small portion of academic engagement is directly influenced by physical exercise behavior, while a larger portion is indirectly affected through physical exercise, which influences self-efficacy and subsequently impacts academic engagement.

Thus, college students need to maintain a high level of self-efficacy while engaging in their studies, set appropriate learning goals, and work toward those goals. Students should also prioritize their physical and mental well-being. Regular physical exercise can help students maintain a positive learning attitude, enhancing their focus and increasing their engagement in their studies.

### 4.3 Limitations and future assumptions

First, this study only reveals the correlational relationships between physical exercise, self-efficacy, and academic engagement without making direct causal inferences. Future research could benefit from adopting longitudinal or experimental designs to better explore the causal relationships between these variables. Second, the scales used are subjective and may have response bias. Third, the participants in this study were limited to university students in Fuzhou, which restricts the generalizability of the findings. Future studies should consider using more diverse sample populations and cross-cultural research to enhance the broader applicability of the results. Fourth, the sample size in this study was relatively small, and the sample selection was somewhat limited. Future research should aim to expand the sample size and include a more diverse range of participants to improve the representativeness and generalizability of the findings.

## 5 Conclusion

This study found positive correlations among physical exercise behavior, academic engagement, and self-efficacy among non-PE majors in Fuzhou City. Physical exercise positively predicts academic engagement and self-efficacy, with self-efficacy mediating the relationship between exercise and engagement. Self-efficacy mediates the relationship between physical exercise behavior and academic engagement.

## Data Availability

The original contributions presented in the study are included in the article/supplementary material, further inquiries can be directed to the corresponding author.
